# Convergent antibody responses are associated with broad neutralization of hepatitis C virus

**DOI:** 10.3389/fimmu.2023.1135841

**Published:** 2023-03-24

**Authors:** Nicole E. Skinner, Clinton O. Ogega, Nicole Frumento, Kaitlyn E. Clark, Harry Paul, Srinivasan Yegnasubramanian, Kornel Schuebel, Jennifer Meyers, Anuj Gupta, Sarah Wheelan, Andrea L. Cox, James E. Crowe, Stuart C. Ray, Justin R. Bailey

**Affiliations:** ^1^ Center for Vaccines and Immunity, The Abigail Wexner Research Institute, Nationwide Children’s Hospital, Columbus, OH, United States; ^2^ Department of Medicine, College of Medicine, The Ohio State University, Columbus, OH, United States; ^3^ Division of Infectious Diseases, Department of Medicine, Johns Hopkins University School of Medicine, Baltimore, MD, United States; ^4^ Department of Oncology, Johns Hopkins University School of Medicine, Baltimore, MD, United States; ^5^ Department of Pathology, Microbiology and Immunology, Vanderbilt University Medical Center, Nashville, TN, United States; ^6^ Department of Pediatrics, Vanderbilt University Medical Center, Nashville, TN, United States; ^7^ Vanderbilt Vaccine Center, Vanderbilt University Medical Center, Nashville, TN, United States

**Keywords:** hepatitis C virus, B cell, neutralizing antibody, B cell receptor, vaccine

## Abstract

**Introduction:**

Early development of broadly neutralizing antibodies (bNAbs) targeting the hepatitis C virus (HCV) envelope glycoprotein E2 is associated with spontaneous clearance of infection, so induction of bNAbs is a major goal of HCV vaccine development. However, the molecular antibody features important for broad neutralization are not known.

**Methods:**

To identify B cell repertoire features associated with broad neutralization, we performed RNA sequencing of the B cell receptors (BCRs) of HCV E2-reactive B cells of HCV-infected individuals with either high or low plasma neutralizing breadth. We then produced a monoclonal antibody (mAb) expressed by pairing the most abundant heavy and light chains from public clonotypes identified among clearance, high neutralization subjects.

**Results:**

We found distinctive BCR features associated with broad neutralization of HCV, including long heavy chain complementarity determining region 3 (CDRH3) regions, specific VH gene usage, increased frequencies of somatic hypermutation, and particular VH gene mutations. Most intriguing, we identified many E2-reactive public BCR clonotypes (heavy and light chain clones with the same V and J-genes and identical CDR3 sequences) present only in subjects who produced highly neutralizing plasma. The majority of these public clonotypes were shared by two subjects who cleared infection. A mAb expressing the most abundant public heavy and light chains from these clearance, high neutralization subjects had features enriched in high neutralization clonotypes, such as increased somatic hypermutation frequency and usage of *IGHV1-69*, and was cross-neutralizing.

**Discussion:**

Together, these results demonstrate distinct BCR repertoires associated with high plasma neutralizing capacity. Further characterization of the molecular features and function of these antibodies can inform HCV vaccine development.

## Introduction

Hepatitis C virus (HCV) is a dire global health problem, with an estimated 1.5 million new infections each year and 58 million people living with chronic HCV, a condition which can lead to liver cirrhosis and hepatocellular carcinoma (1). Although a highly effective cure exists in the form of direct acting antivirals (DAAs), the ability of DAAs to curb the HCV epidemic is hampered by limited accessibility, underdiagnosis, and reinfection following cure (2, 3). A prophylactic vaccine is urgently needed to help achieve the World Health Organization’s goal of eliminating HCV as a public health problem by 2030 (1).

Approximately 25% of adults spontaneously clear HCV infection, while 75% develop persistent infection (4). The factors permitting spontaneous clearance of HCV are multifaceted, with important contributions from anti-viral T cells (5–8) as well as broadly neutralizing antibodies (bNAbs) (9–13), defined as antibodies that target epitopes conserved on genetically diverse viruses. In humanized mouse and primate models, bNAb infusion protects against acquisition of HCV (14–16). In humans, early development of high titers of bNAbs is associated with spontaneous clearance (10, 11, 17), likely by driving evolution of their target, the HCV envelope glycoprotein E2, to an unfit state (9, 18). However, bNAb determinants in HCV are incompletely understood. Greater knowledge of the critical molecular features and epitopes of protective bNAbs is still needed, as vaccines that were designed to target HCV envelope proteins have failed thus far to induce high titers of bNAbs (19–21).

We performed an in-depth analysis of the B cell receptors (BCRs) of HCV E2-reactive B cells during acute infection in people with either high or low plasma neutralizing breadth. We found that subjects who produced highly neutralizing plasma shared multiple BCR repertoire features such as longer heavy chain complementarity determining region 3 (CDRH3) length, skewing toward certain *V_H_* genes (including *IGHV1-69*), higher frequency of somatic hypermutation of the *V_H_*, and even shared *V_H_* gene substitutions. Furthermore, we identified a strikingly large number of public clonotypes, which are genetically similar clones of antibodies, shared among subjects with highly neutralizing plasma. The majority of public clonotypes were shared between subjects with broad plasma neutralizing activity who also spontaneously cleared infection. Finally, in a proof-of-concept experiment, we produced a cross-reactive neutralizing monoclonal antibody (mAb) by pairing the most abundant public heavy and light chains from two subjects with highly neutralizing plasma who cleared infection. This mAb shared many features enriched in high neutralization clonotypes, such as increased somatic hypermutation frequency and usage of *IGHV1-69*. The mAb was also able to neutralize multiple diverse HCV strains. Together, these data demonstrate key features of broadly neutralizing antibody responses and suggests that identification of public BCR clonotypes from individuals who broadly neutralize HCV can reveal important insights into protective anti-HCV bNAb responses.

## Materials and methods

### Study subjects

PBMC and plasma samples from HCV-infected individuals were obtained from the Baltimore Before and After Acute Study of Hepatitis (BBAASH) cohort (22–24). This cohort was established at Johns Hopkins Medicine more than two decades ago and is a prospective cohort of injection drug users in Baltimore who are followed from before infection with HCV, through spontaneous clearance/persistence of HCV over as many as 15 years. Subjects were excluded from use in this study if they were co-infected with HIV or hepatitis B virus. Samples were taken from all subjects during the acute phase of infection and prior to HCV clearance in subjects who spontaneously cleared infection. All subjects were enrolled prior to HCV infection as defined by negative HCV antibody and HCV RNA testing. Subjects were sampled approximately once per month and the onset of infection was determined to be the date halfway between the most recent negative testing and first positive testing. The acute phase of infection was defined as the first 6 months of infection (25). The study was approved by the Institutional Review Board of Johns Hopkins Hospital, and informed written consent was obtained from all study participants.

### HCV viral load and clearance/persistence determinations

HCV viral loads (IU/mL) were quantified after RNA extraction with the use of commercial real-time reagents (Abbot HCV Real-time Assay) migrated onto a research-based real-time PCR platform (Roche 480 LightCycler). HCV seropositivity was determined using the Ortho HCV version 3.0 ELISA Test System (Ortho Clinical Diagnostics). HCV clearance was defined as undetectable HCV RNA for a period of at least 60 days with no recurrence of viremia in individuals with detectable anti‐HCV antibodies. Persistence was defined as detectable HCV RNA viremia for more than 1 year. Day 0 of infection was estimated as the midpoint date of the last negative and first positive HCV RNA test. Duration of infection was calculated as that timepoint’s date minus the date of day 0.

### HCVpp neutralization assay and high/low neutralization capacity determinations

HCVpp were produced by lipofectamine-mediated transfection of HCV E1E2, pNL4-3.Luc.R-E, and pAdVantage (Promega) plasmids into HEK293T cells as previously described (26, 27). Neutralization assays were performed as described previously (28). mAbs at 10 or 100 μg/mL, or heat-inactivated plasma samples at 1:100 dilution were incubated with HCVpp for 1 hour at 37°C prior to addition to Hep3B cells in duplicate. Medium was changed after 5 hours, and cells were incubated for 72 hours before measuring luciferase activity in cell lysates in relative light units (RLUs). Only HCVpp preparations producing RLU at least 10-fold above values of mock pseudoparticles were used for neutralization experiments, and HCVpp input was normalized to 1 × 10^6^ to 6 × 10^6^ RLUs. Nonspecific human IgG (Sigma-Aldrich) was used as a negative control. To quantify neutralization capacity, integrating both breadth and potency, we used a neutralization score (29) whereby plasma neutralization potency was scored for each HCVpp (>80% neutralization received a score of 3, 50-80% received a score of 2, 20-49% received a score of 1, and <20% received a score of 0) and then summed over all HCVpp, using a previously characterized panel of 19 HCVpp representing diverse HCV E1E2 (10). We defined high or low neutralization subjects as subjects with neutralization scores > 10 or < 10, respectively.

For 50% inhibitory concentration (IC_50_) determinations, mAbs were serial 5-fold diluted, starting at a concentration of 100 μg/mL (with phosphate-buffered saline [PBS] only in the last well), and incubated with HCVpps as above. The percentage of neutralization was calculated as [1 − (RLU_mAb_/RLU_PBS_)] × 100, with the PBS RLU values averaged across six values. IC_50_ values were calculated from neutralization curves fit by nonlinear regression (log[inhibitor] *vs* normalized response, variable slope) in Prism 8 software (GraphPad Software, San Diego, CA). mAb-HCVpp tests that did not reach 50% inhibition were assigned an IC_50_ of 100 μg/mL.

### Generation of soluble E2 for B cell sorting

Genes encoding E2 ectodomains of three genotype 1 HCV strains (1a157, 1b09, 1b21) were cloned from a previously described library of E1E2 clones (30) into a mammalian expression vector (phCMV3-Ig Kappa-HIS, a gift of Leopold Kong, The Scripps Research Institute, La Jolla, California, USA). The vector allows expression of soluble E2 (sE2) protein with a C-terminal His tag as well as an N-terminal murine Ig Kappa leader signal for efficient protein secretion. sE2 expression and purification was performed as previously described (9).

### Cell staining and flow cytometric cell sorting

Cell staining of E2-reactive and non-reactive B cells was performed as previously described (31). Briefly, PBMCs were isolated from blood using a Ficoll separation gradient. 30-50 × 10^6^ PBMCs were incubated with anti-CD81 antibody (BD Cat #555675) at 5 µg/ml and Fc blocker (BD Cat #564220) diluted in FACS Buffer (1x PBS with 1% BSA) for 30 minutes at 4°C. Cells were then washed twice with FACS buffer. sE2 cocktail (1a157, 1b09, and 1b21) was added to the cells at 5 µg/mL and incubated at 4°C for 30 minutes. Cells were washed three times with FACS buffer. Fluorophore conjugated antibody cocktail containing CD10-PE (BD Cat #555375), CD19-BV421 (BD Cat #562440), CD3-APC H7 (BD Cat#560176, IgM-BB515 (BD Cat #564622), IgD-BB515 (BD CAT #565243), and Anti His-AF647 (Thermo Fisher Cat #MA1-21315-A647) was added to the cells. They were incubated at 4°C for 30 minutes then washed three times. The viability dye propidium iodide (PI) was added immediately prior to sorting. Cells were sorted using a MoFlo Legacy cell sorter (Beckman Coulter). Mature, class-switched B cells were gated as follows: lymphocytes (FSC by SSC), singlets (FSC by pulse width), live cells (PI), IGM-, IgD-, CD3-, CD10-, CD19+. E2-reactive and E2-nonreactive B cells were sorted directly into cell lysis buffer (Qiagen). PBMCs from at least one healthy control subject were stained with each cell sorting experiment and used for setting of the E2-reactive gate. Nine to ten thousand E2-nonreactive cells were collected, and all E2-reactive cells present were collected (316 to 11,613, median 1,461).

Determination of E2-reactive frequency was done using FlowJo software. Lymphocytes were downsampled to 500,000 so that cell numbers from all HCV subjects and healthy controls would be comparable. Some HCV subjects were sorted multiple times to increase yield for RNA sequencing and their E2-reactive frequencies were averaged.

### Isolation of RNA, library preparation, and sequencing

RNA was isolated using the RNeasy Micro Kit (Qiagen) according to the manufacturer’s instructions. Briefly, cells in lysis buffer containing 2-mercaptoethanol were combined with 70% ethanol and applied to a purification column. Following multiple washes and the application of DNAse, RNA was eluted in RNAse-free water. RNA quality was verified using a 2100 Bioanalyzer (Agilent). cDNA libraries were produced using the SMARTer Human BCR IgG IgM H/K/L Profiling Kit (Takara) according to the manufacturer’s instructions and with the addition of unique molecular identifiers (UMIs). AMPure XP beads (Beckman) were used for purification steps. cDNA library quality was verified using a 2100 Bioanalyzer (Agilent) and quantified using a Qubit (Thermo Fisher). All sequencing was performed on an Illumina MiSeq at a depth of 1 million reads. BCR sequences were validated and identified using MiXCR software (32). Only sequences with nucleotides with Phred quality scores > 20 were included, however subsequent sequences with nucleotides with lower quality scores could be matched to existing clonotypes if the number of low quality nucleotides was < 0.7%. A clonotype was defined by unique V-gene, J-gene, and CDR3 amino acid sequence. All additional analysis was done in R Studio (version 4.0.2).

### Antibody synthesis and ELISA

Heavy chain variable sequences were synthesized (Twist Biosciences) and cloned into a human IgG1 expression plasmid (pTwist CMV BetaGlobin WPRE Neo_IgG1Fc). Kappa and lambda variable sequences were synthesized and cloned into a human IGK or IGL expression plasmid (pTwist CMV BetaGlobin WPRE Neo_Kappa_TAG or pTwist CMV BetaGlobin WPRE Neo_Lambda_TAG). Expi293 cells were transfected with equivalent concentrations of the heavy and light chain vectors using the Gibco Expi293 Expression System, as described by the vendor (Thermo Fisher Scientific). Ig was harvested and purified using Pierce™ Protein G Agarose (Cat. #20398) and buffer-exchanged into PBS, then concentrated using Pierce™ Protein Concentrator PES, 30K MWCO (Cat. #88529).

For E2 binding assays, Immulon 2b microtiter plates were coated with Lectin followed by incubation with sE2 antigen (50 µL at 1 µg/mL) overnight. The next day, the plates were blocked with PBS-TMG (PBS + 0.5% Tween 20 + 1% non-fat dry milk + 1% goat serum) and then incubated with serial dilutions of our experimental mAb, a positive control mAb (HEPC74), or a negative control antibody (human IgG). Anti-human IgG-HRP (BD-Pharmingen Cat #555788) was used at 1:4000 dilution with TMB peroxidase substrate and 1N sulfuric acid to stop the reaction. Plates were read at an absorbance of 450 nm.

### Statistical analysis

All statistical tests were performed in R Studio. Two group comparisons were performed with t tests if data were normally distributed (based on the Shapiro Wilk normality test) or Mann Whitney rank test if data were not normally distributed. Multi-group comparisons were performed using one-way ANOVA if data were normally distributed or Kruskal-Wallis test if data were not normally distributed. *Post hoc* pairwise comparisons were done with the Dunn test. Comparisons of proportions were done using Fisher’s exact test. In all cases where more than one comparison was made, values were adjusted for multiple comparisons using the Benjamini-Hochberg method.

### Study approval

This research was approved by the Johns Hopkins University School of Medicine’s Institutional Review Board (IRB). Prior to blood collection, all participants provided informed written consent.

## Results

### Selection of subjects

**Figure 1:**
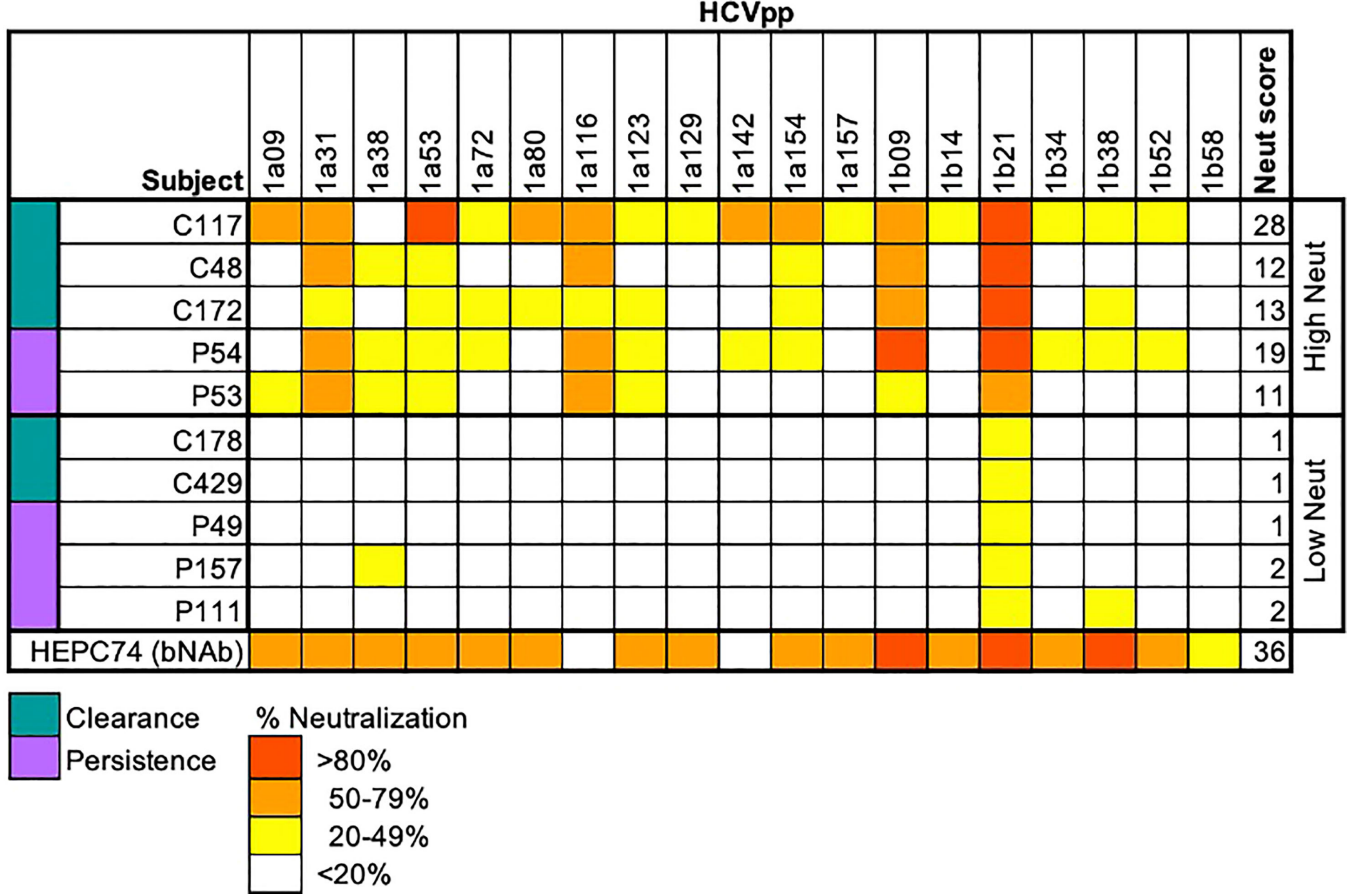
Neutralization score by subject. Percent neutralization achieved by a 1:100 dilution of plasma for each subject was measured using a diverse panel of 19 genotype 1 HCVpp. Values are means of two independent experiments performed in duplicate. As a positive control, the known HCV monoclonal bNAb HEPC74 tested at 10 μg/mL concentration is shown in the bottom row. Neutralization score integrating neutralization potency and breadth is shown in the last column, with the score equaling the sum of the potency for each HCVpp (>80% = 3, 50-79% = 2, 20-49% = 1, <20% =0). High neutralization was defined as neutralization score > 10.

We tested the neutralizing breadth of acute infection plasma samples of 10 subjects (**Table 1**; **Supplemental Table 1**) from the Baltimore Before and After Acute Study of Hepatitis (BBAASH) cohort who had demonstrated either relatively high or low plasma neutralizing breadth in prior testing (33), using a well-characterized panel of 19 genotype 1 HCV pseudoparticles (HCVpp) (10). Subjects were classified as demonstrating high or low neutralization capacity based on their neutralization score, a previously published metric integrating neutralization potency and breadth (29). We included subjects who spontaneously cleared infection and subjects with persistent infection and found high and low neutralization breadth in both groups (**Figure 1**; **Supplemental Table 1**). Subjects with high neutralization capacity (neutralization score > 10) had a longer duration of infection than subjects with low neutralization capacity (**Table 1**), as expected based on prior studies showing a correlation between duration of infection and development of neutralizing antibodies (10, 11, 34). It is important to note that two subjects cleared without producing broadly neutralizing plasma. We hypothesize that clearance in these subjects was mediated by cellular immunity, given the known importance of T cells in spontaneous clearance of HCV (35).

**Table 1:** Subject characteristics.

	High Neut (*n*=5)	Low Neut (*n*=5)	Significance
Age, years^1^			
** Median**	28	27	*ns*
** Range**	(21, 31)	(24, 32)	
Sex^2^			
** Male, n (%)**	3 (60)	3 (60)	*ns*
Race^2^			
** White, n (%)**	5 (100)	5 (100)	*ns*
HCV Genotype^2^			
** 1a (%)**	2 (40)	3 (60)	*ns*
** 1b (%)**	2 (40)	0 (0)	*ns*
** 2b (%)**	0 (0)	1 (20)	*ns*
** 3a (%)**	1 (20)	1 (20)	*ns*
Duration of Infection (Days)^3^			
** Median**	344	108	*p* = 0.02
** Range**	(285, 379)	(82, 337)	
HCV RNA (log_10_ IU/mL)^2^			
** Median**	6.3	5.0	*ns*
** Range**	(4.4, 6.5)	(1.4, 6.5)	

1. Welch’s T-test was used for age comparisons.

2. Fisher’s Exact test was used for sex, race, and HCV genotype comparisons.

3. Mann-Whitney test was used for duration of infection and HCV RNA comparisons.

ns, not significant.

Clearance subjects had lower levels of HCV viremia compared to persistence subjects, as expected, since they were sampled at timepoints nearing clearance. There were no statistically significant differences in age, sex, or infecting HCV genotype between groups. However, all subjects were white and between the ages of 20 and 32 years old due to the demographics of the BBAASH cohort. None of the subjects were co-infected with HIV or hepatitis B virus.

### Frequencies of E2-reactive B cells correlate with neutralization capacity

**Figure 2:**
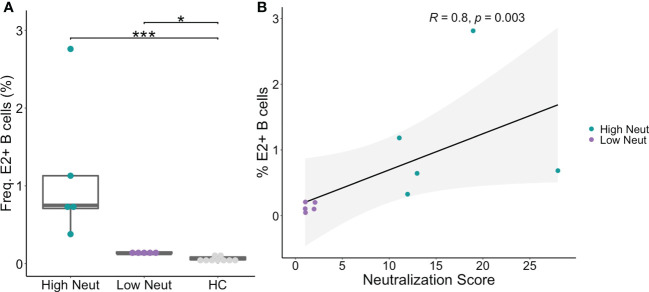
Frequency of E2-reactive B cells. **(A)** Frequency of HCV sE2+ mature, class-switched B cells (% E2+ of CD3-, IgM-, IgD-, CD10-, CD19+, live, singlet lymphocytes) is shown for high and low neutralization subjects. Statistical comparisons were made using the Kruskal-Wallis test followed by the Dunn *post-hoc* test with the Benjamini-Hochberg correction applied for multiple comparisons. Boxplots indicate the 25th percentile (lower border), 75th percentile (upper border), median (horizontal line), and maximum and minimum values that fall within 1.5x the interquartile range (whiskers). **(B)** The frequency of HCV sE2+ mature, class-switched B cells was plotted against plasma neutralization score for each subject. A small amount of random variation (1.5%) was introduced into the location of overlapping data points to ensure all points were visible. *R*, Kendall rank correlation coefficient. Lymphocytes were downsampled to 500,000 cells in these analyses so that equivalent numbers of cells in HCV and healthy controls were compared. *P < 0.05; ***P < 0.001.

PBMCs were obtained from subjects at the same timepoints as plasma samples and sorted using flow cytometry to isolate B cells reactive to the HCV E2 envelope glycoprotein. HCV E2-reactivity was determined by binding of class-switched, mature B cells (CD3-, IgD-, IgM-, CD10-, CD19+) to a cocktail of three genotype 1 soluble HCV E2 (sE2) variant proteins (**Figure 2A**). The three variants (1a157, 1b09, 1b21) were selected because they represented low, medium, or high sensitivity to binding of a panel of previously isolated anti-HCV bNAbs (31). HCV E2-nonreactive B cells were also sorted from each subject as a control. The assay was sensitive and specific for detection of E2-reactive B cells in HCV-infected subjects (**Figure 2A**; **Supplemental Figure S1**). Both high and low neutralization groups had a higher frequency of E2-reactive B cells than healthy (HCV uninfected) controls (**Figure 2A**). Across all subjects, there was a strong positive correlation (R = 0.8, p=0.003) between neutralization score and frequency of E2-reactive B cells (**Figure 2B**).

### Distinct V-gene usage patterns are observed in subjects with highly neutralizing plasma

For E2-reactive and nonreactive B cells, we quantified the number of clonotypes using each V-gene for subjects with highly and poorly neutralizing plasma. A clonotype was defined by unique V-gene, J-gene, and CDR3 amino acid sequence. V-gene usage was normalized by the total number of clonotypes in each group. This was done for *V_H_* genes as well as kappa and lambda light chain V-genes (**Supplemental Figure S2**). Significant differences in V-gene usage were defined as a ≥1.5-fold change in usage with an associated p-value < 0.05 after adjustment for multiple comparisons. Comparisons are shown *via* volcano plot for E2-reactive B cell clonotypes from subjects with highly or poorly neutralizing plasma.

**Figure 3:**
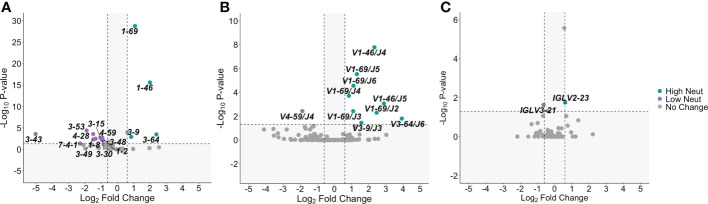
V-gene usage. **(A)** Significant differences in *V_H_* gene expression between E2-reactive high neutralization and low neutralization B cell clonotypes are shown *via* volcano plot. **(B)** Volcano plots show significant differences in *V_H_ – V_J_* gene pair usage between E2-reactive high neutralization and low neutralization B cell clonotypes. **(C)** Significant differences in light chain V-gene expression (*V_K_* and *V_L_* data combined) between E2-reactive high neutralization and low neutralization B cell clonotypes are shown *via* volcano plot. For all volcano plots, significant differences were defined as a minimum 1.5-fold difference with an associated P value < 0.05 after correction for multiple comparisons. (Benjamini-Hochberg method). Dashed vertical lines show the threshold for fold change expressed as Log_2_ Fold Change (0.6 and -0.6). The dashed horizontal line shows the P value threshold expressed as -Log_10_ P-value (1.3). Labeled V-genes meet significance criteria and are colored green to denote increased expression in high neutralization clonotypes or purple to denote increased expression in low neutralization clonotypes. All statistical comparisons were made using Fisher’s exact test with the Benjamini-Hochberg correction for multiple comparisons.

Many different *V_H_* genes differed between E2-reactive B cell clonotypes from subjects with highly and poorly neutralizing plasma (**Figure 3A**). Four *V_H_* genes were enriched in E2-reactive B cell clonotypes from subjects with highly neutralizing plasma (*IGHV1-69*, *IGHV1-46*, *IGHV3-9*, and *IGHV3-64*) while eleven *V_H_* genes were enriched in subjects with poorly neutralizing plasma. *IGHV1-69*, used twice as often in subjects with highly neutralizing plasma (p < 1E-20), is noteworthy both because of its prevalence (>20% of E2-reactive clonotypes from high neutralization subjects use *IGHV1-69*) but also because it is a feature of many previously identified broadly neutralizing antibodies against HCV (36–38).

We also investigated usage of heavy chain V-J gene combinations since these pairings are sometimes enriched in addition to enrichment of individual V-genes. We identified many *V_H_*-*J_H_* gene combinations that were enriched in subjects with highly neutralizing plasma (**Figure 3B**). All the *V_H_* genes enriched in E2-reactive clonotypes from high neutralization subjects were also identified as enriched in at least one *V_H_*-*J_H_* pairing, supporting the significance of these *V_H_* genes. *IGHV1-69* in conjunction with nearly all *J_H_* genes (*IGHJ-2* to *IGHJ-6*) was enriched in high neutralization subjects, whereas some enriched *V_H_* genes were only enriched with a single *V_J_* gene (e.g., *IGHV3-6*/I*GHJ3* and *IGHV3-64*/*IGHJ6*).

Fewer significantly enriched genes were identified in the light chain V-gene analyses (**Figure 3C**). Only one *V_L_* gene was enriched in E2-reactive high neutralization clonotypes (*V_L_2-23*) and low neutralization clonotypes (*IGLV3-21*). No *V_K_* gene frequencies were identified as significantly different. No light chain V-J gene pairings were significantly enriched in E2-reactive B cell clonotypes from either high or low neutralization groups.

### CDRH3 regions are longer in subjects with highly neutralizing plasma

E2-reactive and E2-nonreactive B cells were subjected to bulk RNA sequencing of the variable genes encoding their B cell receptors (BCR-seq). BCR sequences were validated and extracted using MiXCR (32). To account for differences in sequencing depth, we randomly downsampled clonotypes from E2-reactive and E2-nonreactive B cells to obtain equal numbers of clonotypes in each subject.

**Figure 4:**
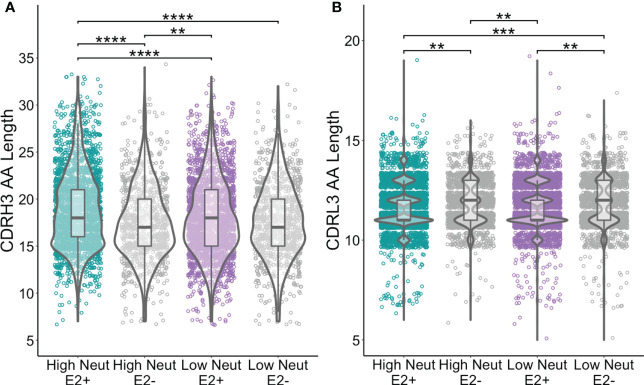
CDR3 length comparisons. **(A)** Comparison of CDRH3 lengths between E2-reactive and non-reactive B cell clonotypes for high and low neutralization subjects. **(B)** Comparison of CDRL3 lengths (IGK and IGL data combined) between E2-reactive and non-reactive B cell clonotypes for high and low neutralization subjects. Statistical comparisons were made using the Kruskal-Wallis test followed by the Dunn *post-hoc* test with the Benjamini-Hochberg correction applied for multiple comparisons. Violin plots show population distributions. Boxplots indicate the 25th percentile (lower border), 75th percentile (upper border), median (horizontal line), and maximum and minimum values that fall within 1.5x the interquartile range (whiskers). **P < 0.01; ***P < 0.001; ****P < 0.0001.

We assessed CDR3 length as a function of neutralization capacity. The amino acid length distribution of CDR3s is often skewed by the response to infection. For instance, bNAbs against HIV often have longer than average CDRH3s (39) and some bNAbs against HCV have also been noted to have long CDRH3s (40). We found that CDRH3s from E2-reactive B cells of subjects with highly neutralizing plasma were longer than CDRH3s from E2-reactive B cells from subjects with poorly neutralizing plasma (mean of 18.9 and 18.0 amino acids long, respectively). CDRH3s from E2-reactive B cells of subjects with highly neutralizing plasma were also slightly longer than CDRH3s from E2-nonreactive B cells of subjects with both highly (17.5 amino acids) and poorly neutralizing (17.6 amino acids) plasma (**Figure 4A**). There was no difference in light chain CDR3 length between E2-reactive B cells from subjects with high and low neutralization scores (**Figure 4B**).

### Conserved CDR1 and CDR2 substitutions are present in BCRs from subjects with highly neutralizing plasma

**Figure 5:**
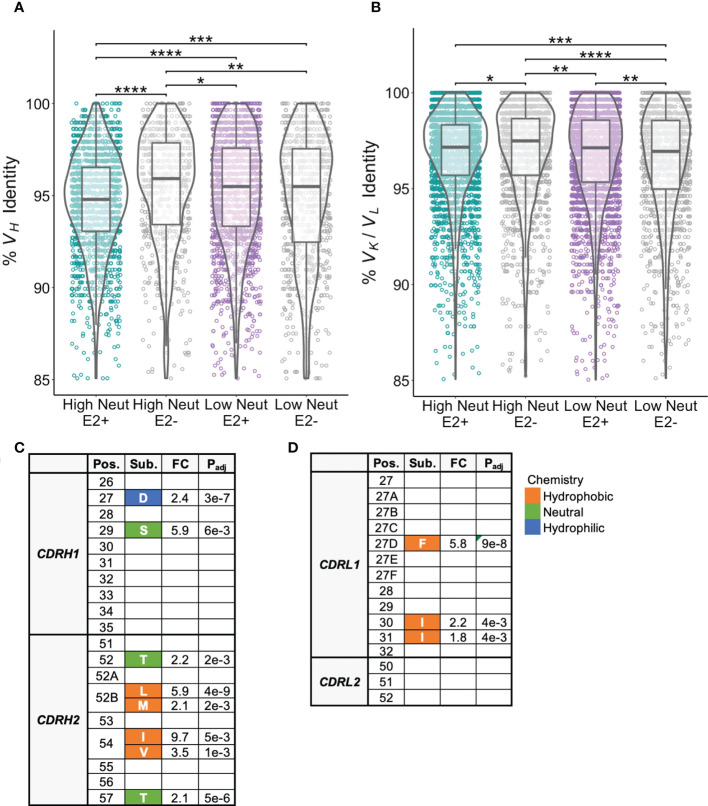
Somatic hypermutation frequencies and shared CDR1 and CDR2 substitutions. **(A)** Somatic hypermutation of the *V_H_* gene expressed as percent identity to germline *V_H_* for E2-reactive and non-reactive B cell clonotypes for high and low neutralization subjects. **(B)** Somatic hypermutation of the light chain V-gene (*V_K_* and *V_L_* data combined) expressed as percent identity to germline *V_K_* or *V_L_* for E2-reactive and non-reactive B cell clonotypes for high and low neutralization subjects. **(C)** CDRH1/CDRH2 substitutions enriched in E2-reactive B cell clonotypes from high neutralization subjects compared to E2-reactive clonotypes from low neutralization subjects. **(D)** CDRL1/CDRL2 substitutions enriched in E2-reactive B cell clonotypes from high neutralization subjects compared to E2-reactive clonotypes from low neutralization subjects. Kabat numbering is used for position. Color indicates hydrophobic (orange), neutral (green), or hydrophilic (blue) amino acid chemistry. Enriched substitutions were defined as those with a minimum 1.5-fold difference between groups with an associated P value < 0.05 after correction for multiple comparisons. For **(A**, **B)**, violin plots show population distributions. Boxplots indicate the 25th percentile (lower border), 75th percentile (upper border), median (horizontal line), and maximum and minimum values that fall within 1.5x the interquartile range (whiskers). Statistical comparisons for **(A**, **B)** were made using the Kruskal-Wallis test followed by the Dunn *post-hoc* test with the Benjamini-Hochberg correction applied for multiple comparisons. Statistical comparisons for **(C**, **D)** were made using Fisher’s exact test with the Benjamini-Hochberg correction for multiple comparisons. Germline V-gene amino acids for each position shown in **(C, D)** can be found in **Supplemental Tables 2, 3**. FC, fold change; P_adj_, adjusted P-value; *P < 0.05; **P < 0.01; ***P < 0.001; ****P < 0.0001.

We next investigated frequencies of somatic hypermutation and accumulation of shared substitutions. E2-reactive clonotypes from subjects with highly neutralizing plasma had higher frequencies of *V_H_* mutation relative to inferred germline nucleotide sequences than those with poorly neutralizing plasma (**Figure 5A**). We suspected these differences might be partially explained by the longer duration of infection in subjects with highly neutralizing plasma compared to poorly neutralizing plasma (**Table 1**), however we saw no correlation between duration of infection and *V_H_* mutation frequencies (**Supplemental Figure S3**). There were no significant differences in light chain V-gene mutation frequencies between E2-reactive clonotypes from subjects with highly and poorly neutralizing plasma (**Figure 5B**).

Since *V_H_* genes from E2-reactive B cell clonotypes from high neutralization subjects were more somatically mutated than those from low neutralization subjects, we hypothesized that we might be able to identify substitutions that were associated with broad neutralizing activity. We focused our analysis on individual E2-reactive B cell *V_H_* substitutions in regions likely to influence antibody binding or function. We aligned CDR1 and CDR2 regions across clonotypes and found multiple CDRH1/CDRH2 substitutions enriched in subjects with highly neutralizing plasma compared to subjects with poorly neutralizing plasma (**Figure 5C**). Consistent with prior studies demonstrating that CDRH2 can play an important role in the recognition of hydrophobic residues in the E2 front layer (40–42), multiple hydrophobic substitutions were enriched at positions 52B and 54 in CDRH2. There were no substitutions enriched in E2-reactive B cell clonotypes in low neutralization subjects.

There were also some light chain CDR1 substitutions enriched in subjects with highly neutralizing plasma compared to subjects with poorly neutralizing plasma. There were no enriched light chain CDR2 substitutions (**Figure 5D**). As with the heavy chain analysis, there were no light chain CDR1 or CDR2 substitutions enriched in low neutralization subjects. Interestingly, although some heavy and light chain substitutions occurred predominantly in one or two V-genes, many substitutions occurred in a variety of different V-genes (**Supplemental Tables 2**-**3**). Unfortunately, due to the high variability in CDR3 length and sequence, it was not possible to reliably align CDR3 sequences to identify enriched CDR3 substitutions.

### HCV E2-reactive public clonotypes were identified from clearance subjects and subjects with high neutralization capacity

Because we were interested in identifying commonalities in BCR sequences favoring the development of bNAbs, we looked for public clonotypes among E2-reactive B cells from high neutralization subjects. We defined a clonotype as public if BCRs with identical CDR3 amino acid sequences and the same V and J-gene usage were found in more than one subject of the same group. We further restricted public clonotypes of interest to group-specific public clonotypes that were shared only among members of a given group but not present in other groups (*e.g*., present in multiple subjects in the high neutralization/E2-reactive group but not present in the low neutralization/E2-reactive or any E2-nonreactive groups), increasing the probability of identifying features unique to HCV neutralization. Since bulk RNA-seq data does not identify heavy and light chain pairs, we identified heavy chain public clonotypes separately from light chain public clonotypes. To ensure that all public clonotypes were identified, we did not downsample clonotypes to normalize by subject for these analyses but expressed number of public clonotypes as the proportion of all clonotypes in the group.

**Figure 6:**
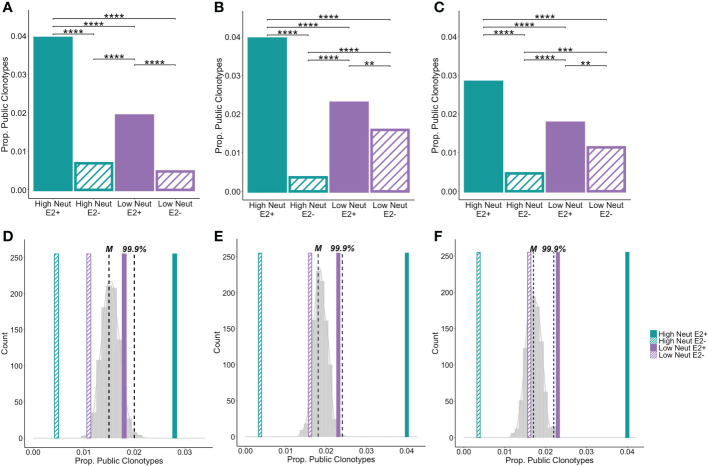
Group-specific public clonotypes. The proportion of group-specific public IGH **(A)**, IGK **(B)**, and IGL **(C)** E2-reactive and non-reactive B cell clonotypes for high and low neutralization subjects. A group-specific public clonotype was defined as having identical CDR3 amino acid sequence and the same V and J-gene usage while being present in at least two members of the same group and not present in any members of other groups. Data are expressed as the proportion of group-specific public clonotypes out of the total number of E2-reactive clonotypes in the group. A histogram shows the frequency of group-specific public clonotype proportions resulting from 1000 trials of random permutation of the IGH **(D)**, IGK **(E)**, and IGL **(F)** E2-reactive clonotype data. Medians are marked by dashed vertical lines labeled with an “M” and the 99.9^th^ percentile is marked with a dashed vertical line labeled “99.9%.” The proportion of group-specific public clonotypes for E2-reactive B cells is marked with solid, color-coded vertical bars (green and purple for high and low neutralization groups, respectively). The proportion of group-specific public clonotypes for E2 non-reactive B cells is marked with hashed, color-coded vertical bars (green and purple for high and low neutralization groups, respectively). Statistical comparison in **(A–C)** were made using Fisher’s exact test with the Benjamini-Hochberg correction for multiple comparisons. **P < 0.01; ***P < 0.001; ****P < 0.0001.

We found that nearly 4% of heavy chain clonotypes of E2-reactive B cells of high neutralization subjects were group-specific public clonotypes (**Figure 6A**). Frequencies of group-specific public clonotypes were significantly lower for E2-reactive B cells of low neutralization subjects (1.9%), and E2-nonreactive B cell subsets (0.05 to 0.07%). These results were mirrored in the light chain analyses, where the proportions of kappa (**Figure 6B**) and lambda (**Figure 6C**) public clonotypes were higher in E2-reactive B cells from high neutralization subjects compared to E2-reactive B cells from low neutralization subjects and E2-nonreactive B cells. For both heavy and light chain analyses, the proportion of public clonotypes in E2-reactive B cells from low neutralization subjects was significantly greater than in E2-nonreactive B cells.

To compare these frequencies with the amount of clonotype sharing that would be expected due to chance, we performed random permutation of data, wherein clonotypes were randomly reassigned to subjects irrespective of group, and the frequency of group-specific public clonotypes for a group of 5 subjects was calculated. This process was performed 1,000 times and a probability distribution of group-specific public clonotype frequencies was generated. The median of this distribution for heavy chain clonotypes was 1.5% with the 99.9^th^ percentile falling at 2%. The frequencies of group-specific public clonotypes for low neutralization E2-reactive B cells as well as high and low neutralization nonreactive B cells fell within this distribution. However, for E2-reactive B cells in high neutralization subjects, frequencies of group-specific public clonotypes fell well above the 99.9^th^ percentile, making it exceedingly unlikely that this degree of clonotype sharing occurred by chance (**Figure 6D**). We performed the same analysis for kappa (**Figure 6E**) and lambda (**Figure 6F**) light chains with similar results, noting only that for the lambda analysis, the frequency of group-specific public clonotypes for the E2-reactive low neutralization group fell just beyond the 99.9^th^ percentile.

Finding such a large number of public clonotypes was surprising given previously published estimates of public clonotypes frequencies being < 1% of the B cell repertoire (43). In addition, finding so many public clonotypes was remarkable given that the overall diversity of clonotypes in each sample was quite high. When we investigated diversity of heavy and light chain clonotypes, we found no statistically significant differences between neutralization groups or between E2-reactive and non-reactive clonotypes (**Supplemental Figure S4**). This was true for two different measures of population diversity, the Shannon diversity and species richness. By both measures, diversity was very high for all subjects, demonstrating that there were very few duplicated clonotypes within subjects. Given the vast diversity of B cell specificities present in individuals (44), this was not surprising; however, it underscores how remarkable it is to find many shared clonotypes between subjects.

In general, the E2-reactive public clonotypes for high neutralization and low neutralization groups were representative of the larger population of high and low neutralization clonotypes, respectively. There were no differences in CDR3 length between the public clonotypes and their respective general populations. There was one *V_H_* (*IGHV1-02*) and one *V_L_* (*IGLV3-10*) gene that were differentially expressed in the high neutralization public clonotypes relative to all high neutralization clonotypes. No V-genes were differentially expressed between low neutralization public clonotypes and all low neutralization clonotypes. Interestingly, public high neutralization light chain clonotypes were more somatically mutated than the non-public clonotypes and low neutralization public light chain clonotypes. There was no difference in *V_H_* somatic hypermutation frequencies between the public and non-public clonotypes (**Supplemental Figure S5**).

### Unbiased clustering of subjects based on clonotype sharing

When we began studying the public clonotypes in more detail, we found that the majority of heavy chain E2-reactive public clonotypes from the high neutralization group were present in two high neutralization subjects who spontaneously cleared infection (C48 and C117). There were no E2-nonreactive public clonotypes between these two subjects, which suggests that the sharing of E2-reactive public clonotypes was driven by similar responses to HCV infection rather than genetic or environmental similarities between these subjects.

**Figure 7:**
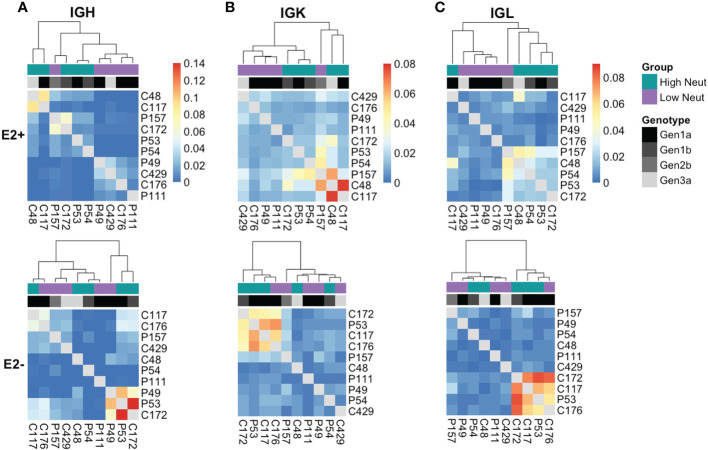
Unbiased clustering of subjects based on public clonotype sharing. Heatmaps showing sharing of IGH **(A)**, IGK **(B)**, and IGL **(C)** clonotypes from E2-reactive (top row) and E2 non-reactive (bottom row) B cells between all subjects. Each column and row are labeled with a subject. Each column also has a colored box to indicate each subject’s neutralization status as well as a grayscale box to indicate each subject’s infecting HCV genotype. The ordering of subjects is determined by average-link clustering with the resultant dendrogram shown at the top of each heatmap.

To determine in an unbiased way whether neutralization capacity was a driver of clonotype sharing, we quantified the number of shared clonotypes between each pair of subjects and performed average-link clustering to reveal relationships among subjects. When analyzing sharing of heavy chain clonotypes between E2-reactive B cells (**Figure 7A**, top), we found that subjects clustered by their neutralization status rather than by their clearance status, with only one outlier (subject P157). However, the highest degree of sharing occurred between C48 and C117, as expected based on our analysis of group-specific public clonotypes. Subjects did not cluster by infecting HCV genotype, suggesting this was not a primary driver of public clonotypes in E2-reactive B cells, which is consistent with prior publications showing that E1E2 antigenicity is not dictated by genotype (34, 45). Notably, C48 and C117 were infected with genotype 3a and 1a virus, respectively. In contrast, E2-nonreactive B cell clonotype clustering occurred without segregation by neutralization capacity and appeared more strongly related to infecting HCV genotype (**Figure 7A**, bottom).

Analysis of kappa light chain shared clonotypes among E2-reactive B cells similarly showed clustering predominantly by neutralization capacity, with C48 and C117 sharing the most of any two subjects (**Figure 7B**, top). Kappa light chain E2-nonreactive clonotype sharing similarly did not appear to be related to neutralization capacity (**Figure 7B**, bottom.) Lambda light chain sharing among E2-reactive B cell clonotypes also clustered largely by neutralization capacity (**Figure 7C**, top). Considerable sharing of lambda light chains between C48 and C117 was likewise present; however, sharing between these two subjects was not as pronounced as in the heavy chain and kappa chain analyses, suggesting that most of the public chain clonotypes between these two subjects used kappa light chains. Clustering among lambda light chain E2-nonreactive clonotypes was similarly not driven by neutralization capacity (**Figure 7C**, bottom).

### Antibody production and neutralization analysis

**Figure 8:**
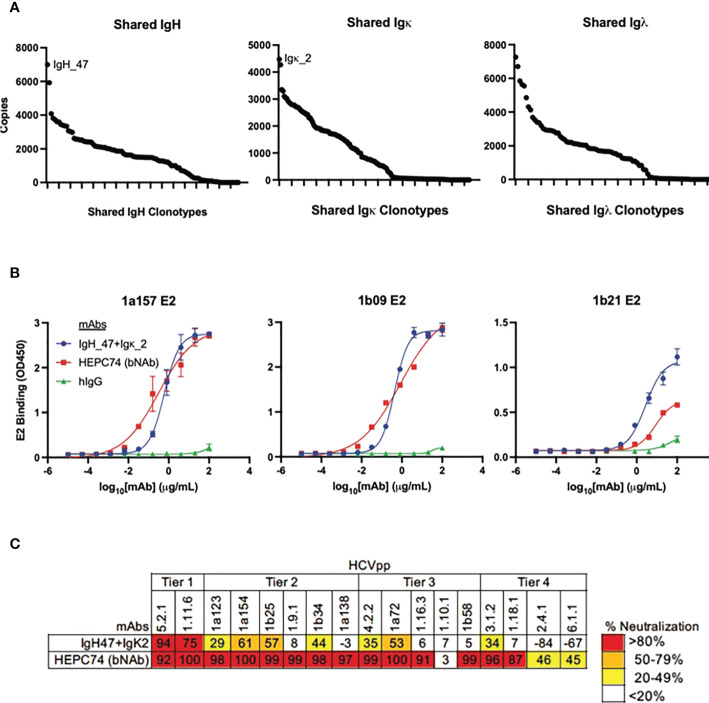
Binding and neutralization assays for public clonotype mAb. **(A)** The abundance of clearance, high neutralization public clonotypes (total number of times a sequence was observed) is shown for IGH (left), IGK (middle), and IGL (right). The most abundant IGH public clonotype (designated IGH_47) and the most abundant light chain public clonotype (designated IGK_2) present in subjects C117 and C48 were cloned into IgG1 and IgK expression vectors and co-transfected to generate a mAb. **(B)** HCV E2 binding ELISA of the public clonotype mAb (IGH_47 + IGK_2, blue) using 1a157 (left), 1b09 (middle), and 1b21 (right) E2 variant proteins. The HEPC74 bNAb (red) was used as a positive control and non-reactive human IgG (green) was used as a negative control. **(C)** Neutralization breadth of the public clonotype mAb (IGH_47 + IGK_2) measured at 100 μg/mL concentration using a panel of 17 genotype 1-6 HCVpp selected to span 4 tiers of increasing neutralization resistance. Values are the average of duplicate wells. As a positive control, the known HCV bNAb HEPC74 tested at 100 μg/mL concentration is shown in the bottom row.

The large number of public clonotypes shared by C48 and C117 (89 IGH, 110 IGK, and 70 IGL clonotypes) were of interest since they derived from subjects at the overlap of clearance and high neutralization capacity. In addition, our prior work on the repertoire of subject C117 revealed that this subject produced broadly neutralizing antibodies associated with evolution of HCV to an unfit state, leading to clearance (9). Consequently, we hypothesized that these shared sequences encoded anti-HCV bNAbs. Because we performed bulk RNA seq analysis of heavy and light chain variable genes separately, we could not determine the authentic pairing of BCR heavy and light chains. However, as a proof of principle experiment, we produced a (mAb) by cloning and expressing the most abundantly expressed public heavy chain and most abundantly expressed public light chain sequences that were shared by these two subjects (**Figure 8A**). Although the most abundant light chains overall were lambda chains, the most abundant light chain shared between C48 and C117 was a kappa chain. This mAb had features we found to be enriched in high neutralization clonotypes and known HCV bNAbs. These included a higher-than-average frequency of somatic mutation of the heavy chain (92% homology to the germline V-gene amino acid sequence) and light chain (94% germline V-gene homology), as well as usage of *IGHV1-69* (**Supplemental Table 4**). The mAb bound to three different variant E2 proteins with an affinity comparable to a control bNAb, HEPC74 (**Figure 8B**). This antibody also exhibited >50% neutralization for 5 of 17 HCVpp in a panel with four tiers of increasing neutralization resistance (45), including both genotype 1 and genotype 5 isolates (**Figure 8C**). We also performed full neutralization curves for HCVpp with detectable neutralization in the initial screen, and confirmed cross-neutralization of two HCVpp (**Supplemental Figure S6**).

## Discussion

We sorted E2-reactive B cells from subjects with acute HCV infection who produced broadly or poorly neutralizing plasma. It is important to note that limitations in available subjects with divergent neutralization capacities and sufficient sample for analysis during acute infection resulted in selection of subjects who lacked demographic diversity. Although subjects were similar demographically between groups, all subjects were young (20-32 years old) and white, which could limit applicability to older individuals and individuals of other races. In addition, we focused only on one time point during acute infection. Although other recent studies have characterized bNAbs predominantly during late chronic infection (46, 47), we restricted our analysis to acute infection with the rationale that immune responses detected during this phase might be more important in promoting HCV clearance. Lastly, we could not identify the presence or absence of interfering antibodies in plasma samples, which could have influenced the measurement of neutralization activity.

In aggregate, our BCR repertoire analyses revealed notable differences in the features associated with broad or poor neutralization of HCV. Broad neutralization was associated with a higher frequency of E2-reactive B cells, longer CDRH3s, a distinct pattern of enriched *V_H_* and *V_K_* genes [including *IGHV1-69*, a gene used by many well-characterized HCV bNAbs (36–38)], increased frequencies of *V_H_* somatic mutation, and multiple enriched CDR1 and CDR2 substitutions, indicative of convergent evolution in both heavy and light chain V-genes. This is consistent with data from other groups suggesting that longer CDRH3, greater frequencies of somatic mutation, and *IGHV1-69* gene usage are important features of HCV bNAbs (46, 47).

Identifying the BCR features associated with bNAb production is important for efforts to produce a prophylactic HCV vaccine. To our knowledge, our BCR sequences are unique, not shared with any published HCV bNAbs. This may be because it is only possible to sample a small portion of the very diverse BCR repertoire. Nevertheless, it is reassuring that our data support the importance of bNAb features identified from previously isolated HCV bNAbs. However, our unbiased, in-depth survey of BCRs in people with broadly neutralizing plasma also revealed new insights, including multiple other *V_H_* genes and one *V_L_* gene associated with broad neutralization as well as specific conserved CDR1 and CDR2 substitutions. It is interesting that many of the CDRH2 conserved substitutions are hydrophobic (4 of 6) since *IGHV1-69* encodes an unusually hydrophobic CDRH2, including at positions 52_B_ (I) and 54 (F/L), which is important for its ability to interact with the predominantly hydrophobic front layer of HCV E2 (37, 48). Furthermore, a recent study characterizing *IGHV1-69*-encoded broadly neutralizing antibodies identified I52_B_M as a substitution associated with increased neutralization capacity in *IGHV1-69*-encoded antibodies. This same study also identified the CDRH1 substitution G27D (47). It is interesting that these same substitutions arose in our analysis which was not restricted to *IGHV1-69*. We also observed that the BCR heavy chain drove most of the differences between groups, a finding consistent with prior bNAb characterization studies (49).

We identified a large number of group-specific public clonotypes shared among E2-reactive B cells from high neutralization subjects. An unbiased clustering analysis showed that overall sharing among subjects was closely correlated with neutralization capacity. In addition, the majority of shared heavy chain and kappa light chain sharing occurred between two high neutralization subjects who also spontaneously cleared infection (C48 and C117). In fact, a remarkably high percentage of clonotypes from C48 and C117 were public clonotypes shared with one another (10% and 16% of heavy chain clonotypes, respectively). To our knowledge, this is the first identification in HCV of public B cell clonotypes meeting this high degree of similarity. A recent detailed analysis of the BCR repertoire in healthy adults showed that the average pair of individuals shared less than 1% of their clonotypes (43). Even when a less stringent definition of public clonotype was applied (not requiring identical CDRH3 sequences), public clonotypes only made up 1-6% of the repertoire (50).

Since these experiments were done using bulk RNA seq analysis, we did not know the authentic pairing of heavy and light chain sequences. However, we synthesized an antibody using the most abundant shared IGH and light (IGK) chains from C48 and C117, hypothesizing that since both subjects cleared HCV and produced neutralizing antibodies, their shared BCR sequences had a high probability of yielding neutralizing antibodies important in HCV clearance. This approach carries significant risk of mispairing of the heavy and light chain. Nevertheless, the antibody produced was functional, binding to diverse HCV E2 proteins and, although not as broadly neutralizing as one of the most broadly neutralizing mAbs isolated against HCV (HEPC74), it was able to neutralize genetically diverse HCVpp.

In the face of rising HCV incidence in the United States, the need for a protective HCV vaccine is urgent. Nevertheless, the necessary features of a protective antibody response are still unknown. These data reveal novel BCR features associated with broad neutralization that arise during acute infection. Further unbiased evaluation of antibody responses in individuals who mount broadly neutralizing antibody responses to HCV is a promising path for characterizing protective humoral immunity to HCV.

## Data availability statement

The datasets presented in this study can be found in online repositories. The names of the repository/repositories and accession number(s) can be found below: PRJNA923033 (SRA). The R code used to analyze the data can be found in a Github repository titled ‘HCV_Neutralization_BCR_analysis’.

## Ethics statement

The studies involving human participants were reviewed and approved by Institutional Review Board of Johns Hopkins Hospital. The patients/participants provided their written informed consent to participate in this study.

## Author contributions

NS, SR and JB conceived and designed the experiments. NS, CO, NF, KC and HP performed experiments. NS analyzed data. SY, KS, JM, AG and SW provided RNA sequencing support and assisted with data analysis. AC, JC, SR and JB provided expert guidance. NS, JB, and JC wrote and edited initial drafts. All authors contributed to the article and approved the submitted version.
