# Corrigendum: Convergent antibody responses are associated with broad neutralization of hepatitis C virus

**DOI:** 10.3389/fimmu.2023.1201033

**Published:** 2023-04-25

**Authors:** Nicole E. Skinner, Clinton O. Ogega, Nicole Frumento, Kaitlyn E. Clark, Harry Paul, Srinivasan Yegnasubramanian, Kornel Schuebel, Jennifer Meyers, Anuj Gupta, Sarah Wheelan, Andrea L. Cox, James E. Crowe, Stuart C. Ray, Justin R. Bailey

**Affiliations:** ^1^ Center for Vaccines and Immunity, The Abigail Wexner Research Institute, Nationwide Children’s Hospital, Columbus, OH, United States; ^2^ Department of Medicine, College of Medicine, The Ohio State University, Columbus, OH, United States; ^3^ Division of Infectious Diseases, Department of Medicine, Johns Hopkins University School of Medicine, Baltimore, MD, United States; ^4^ Department of Oncology, Johns Hopkins University School of Medicine, Baltimore, MD, United States; ^5^ Department of Pathology, Microbiology and Immunology, Vanderbilt University Medical Center, Nashville, TN, United States; ^6^ Department of Pediatrics, Vanderbilt University Medical Center, Nashville, TN, United States; ^7^ Vanderbilt Vaccine Center, Vanderbilt University Medical Center, Nashville, TN, United States

**Keywords:** hepatitis C virus, B cell, neutralizing antibody, B cell receptor, vaccine

In the published article, there was an error in the author list, and author Harry Paul was erroneously excluded. The corrected author list appears below.

Nicole E. Skinner^1,2*^, Clinton O. Ogega^3^, Nicole Frumento^3^, Kaitlyn E. Clark^3^, Harry Paul^3^, Srinivasan Yegnasubramanian^3^, Kornel Schuebel^3^, Jennifer Meyers^3^, Anuj Gupta^3^, Sarah Wheelan^3^, Andrea L. Cox^3^, James E. Crowe, Jr.^4^, Stuart C. Ray^3^, Justin R. Bailey^3*^

In the published article, there was an error. An author was inadvertently excluded from the **Author contributions** section. The corrected **Author contributions** section appears below.

NS, SR and JB conceived and designed the experiments. NS, CO, NF, KC and HP performed experiments. NS analyzed data. SY, KS, JM, AG and SW provided RNA sequencing support and assisted with data analysis. AC, JC, SR and JB provided expert guidance. NS, JB, and JC wrote and edited initial drafts. All authors contributed to the article and approved the submitted version.

The authors apologize for these errors and state that this does not change the scientific conclusions of the article in any way. The original article has been updated.

