# Supplementary material for: Convergent antibody responses are associated with broad neutralization of hepatitis C virus
Source: Front Immunol. 2023 Mar 24;14:1135841. doi: 10.3389/fimmu.2023.1135841 (PMC10080129; doi:10.3389/fimmu.2023.1135841)
Supplement: Supplementary file 1 [file Image_1.pdf]

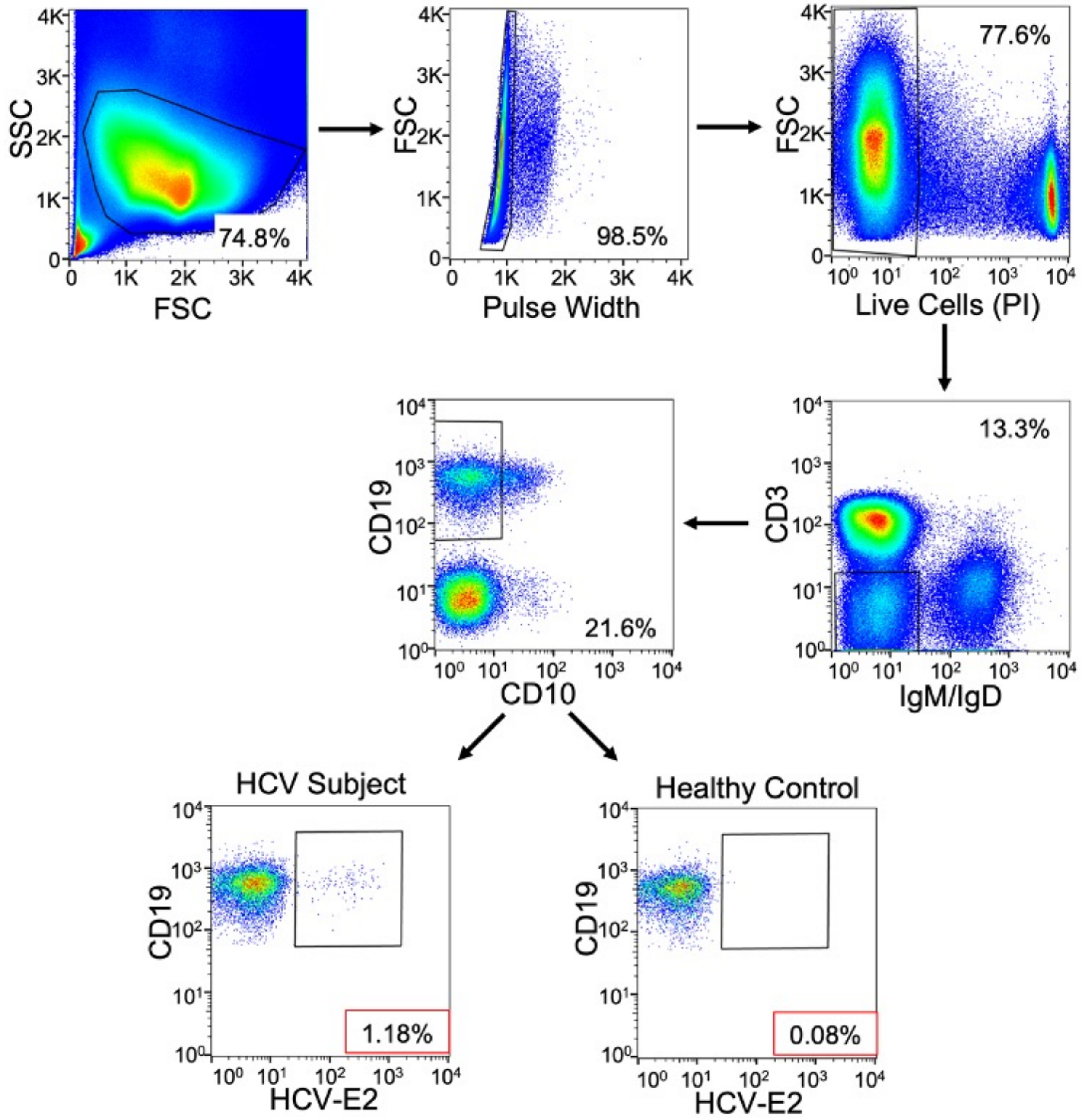

Supplemental Figure S1. Flow cytometry gating strategy. The flow cytometric gating strategy is shown for staining and sorting of E2-reactive B cells. Final gating of HCV sE2+ cells for a representative HCV subject is shown in comparison to a healthy control.
