# Supplementary material for: Convergent antibody responses are associated with broad neutralization of hepatitis C virus
Source: Front Immunol. 2023 Mar 24;14:1135841. doi: 10.3389/fimmu.2023.1135841 (PMC10080129; doi:10.3389/fimmu.2023.1135841)
Supplement: Supplementary file 2 [file Image_2.pdf]

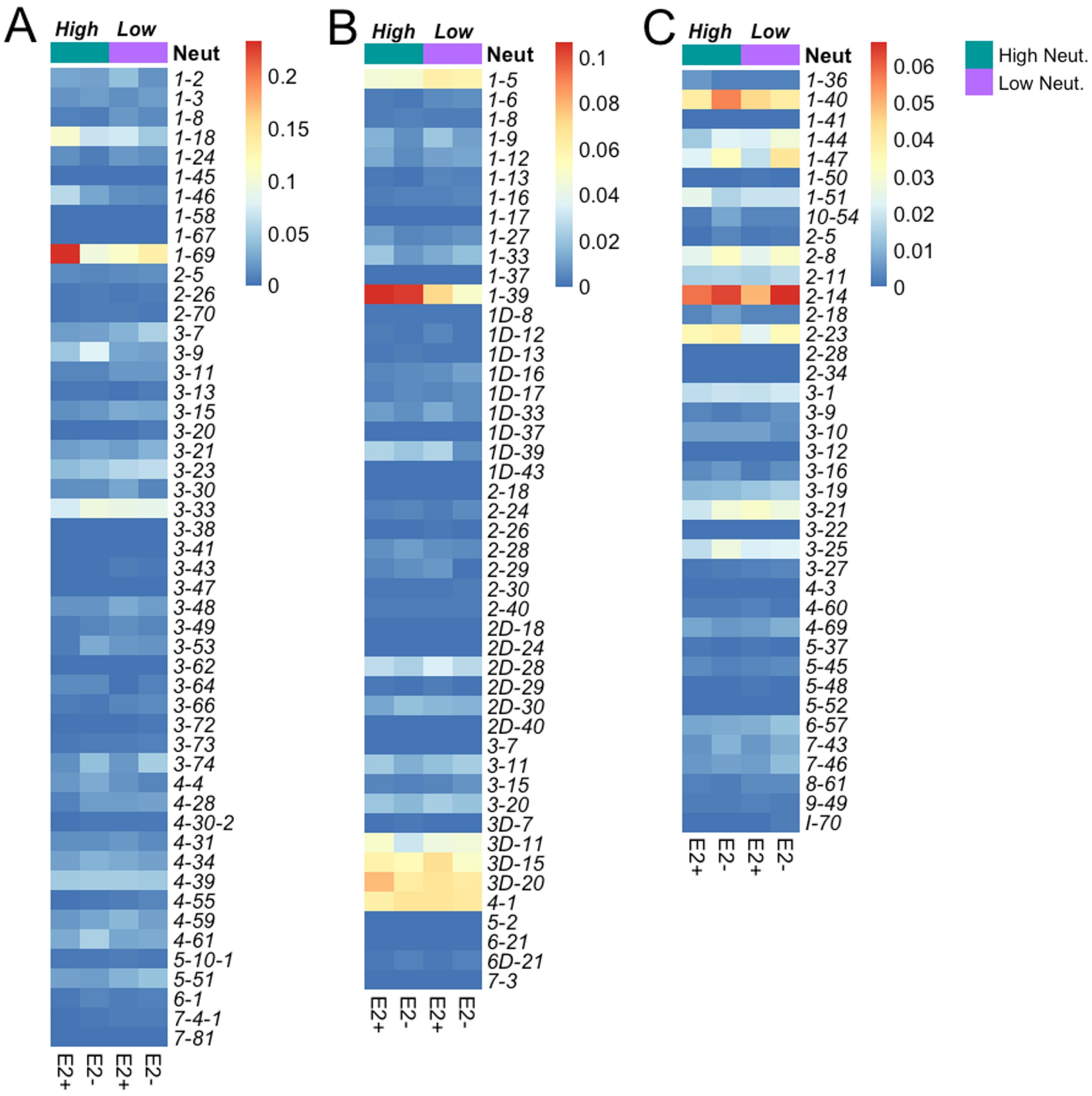

Supplemental Figure S2. (A) Heatmap showing V_H gene usage of E2-reactive and non-reactive B cell clonotypes for high and low neutralization subjects. **(B)** Heatmap showing V_K gene usage of E2-reactive and non-reactive B cell clonotypes for high and low neutralization subjects. **(C)** Heatmap showing V_L gene usage of E2-reactive and non-reactive B cell clonotypes for high and low neutralization subjects. Usage of a given V_H, V_K, or V_L gene is expressed as a proportion of total light chain gene usage by the group.
