# Supplementary material for: Convergent antibody responses are associated with broad neutralization of hepatitis C virus
Source: Front Immunol. 2023 Mar 24;14:1135841. doi: 10.3389/fimmu.2023.1135841 (PMC10080129; doi:10.3389/fimmu.2023.1135841)
Supplement: Supplementary file 3 [file Image_3.pdf]

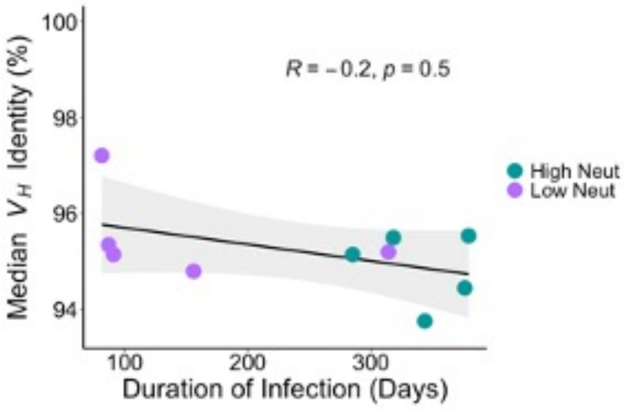

Supplemental Figure S3. V_H somatic hypermutation frequencies as a function of duration of infection. The median V_H somatic mutation frequency (expressed as percent identity to germline V_H) is plotted against duration of infection in days for each subject. High neutralization and low neutralization subjects are colored green and purple, respectively. *R*, Kendall rank correlation coefficient.
