# Supplementary material for: Convergent antibody responses are associated with broad neutralization of hepatitis C virus
Source: Front Immunol. 2023 Mar 24;14:1135841. doi: 10.3389/fimmu.2023.1135841 (PMC10080129; doi:10.3389/fimmu.2023.1135841)
Supplement: Supplementary file 4 [file Image_4.pdf]

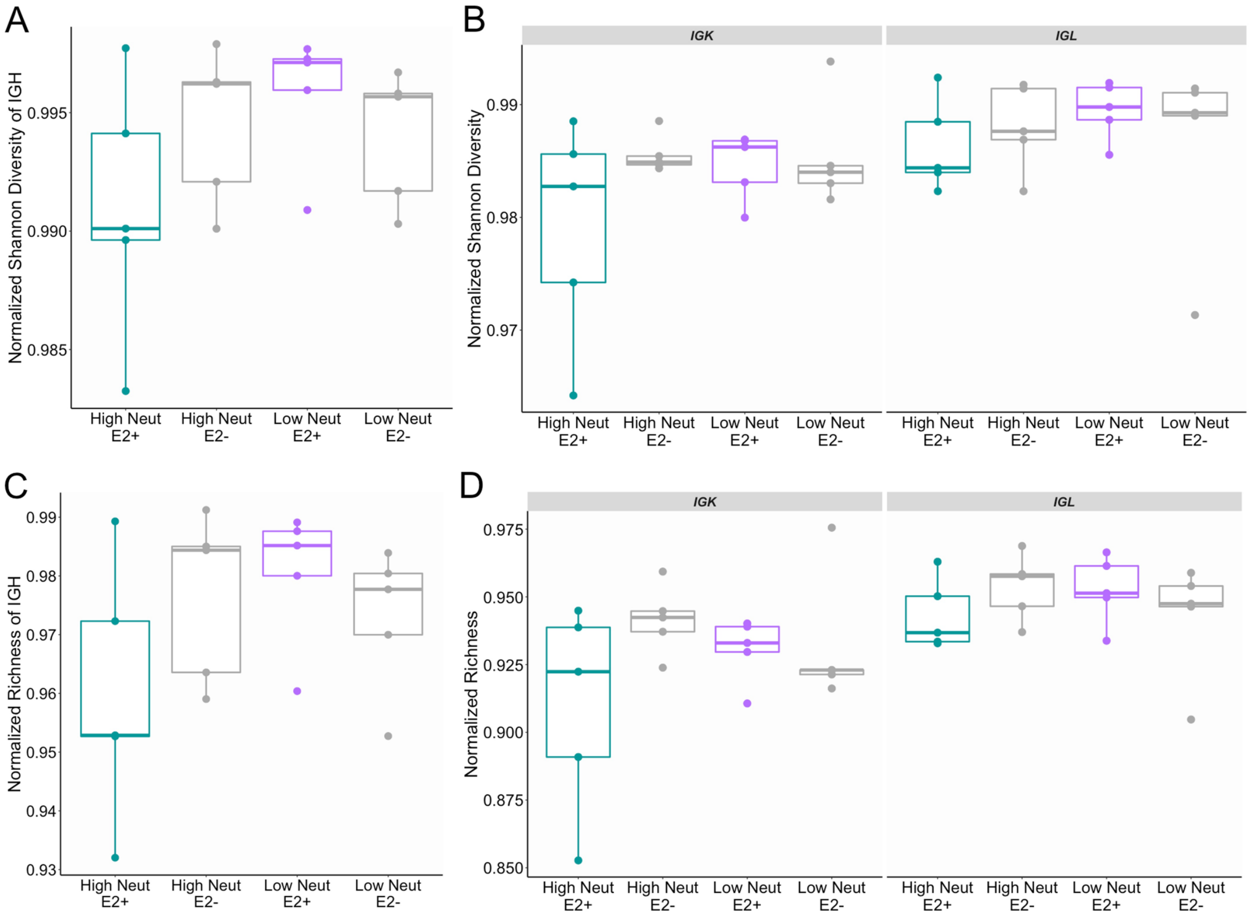

Supplemental Figure S4. Clonotype diversity comparisons. (A) Normalized Shannon diversity of IGH clonotypes is shown for E2-reactive and non-reactive B cells from high and low neutralization subjects. **(B)** Normalized Shannon diversity of IGK (left panel) and IGL (right panel) clonotypes is shown for E2-reactive and non-reactive B cells from high and low neutralization subjects. **(C)** Normalized species richness for IGH clonotypes is shown for E2-reactive and non-reactive B cells from high and low neutralization subjects. **(D)** Normalized species richness of IGK (left panel) and IGL (right panel) clonotypes is shown for E2-reactive and non-reactive B cells from high and low neutralization subjects. Normalization was done by dividing the diversity measurement (Shannon diversity or species richness) by the maximum diversity possible for each subject. Boxplots indicate the 25th percentile (lower border), 75th percentile (upper border), median (horizontal line), and maximum and minimum values that fall within 1.5× the interquartile range (whiskers). Statistical comparisons were made using the Kruskal-Wallis test.
