# Supplementary material for: Convergent antibody responses are associated with broad neutralization of hepatitis C virus
Source: Front Immunol. 2023 Mar 24;14:1135841. doi: 10.3389/fimmu.2023.1135841 (PMC10080129; doi:10.3389/fimmu.2023.1135841)
Supplement: Supplementary file 5 [file Image_5.pdf]

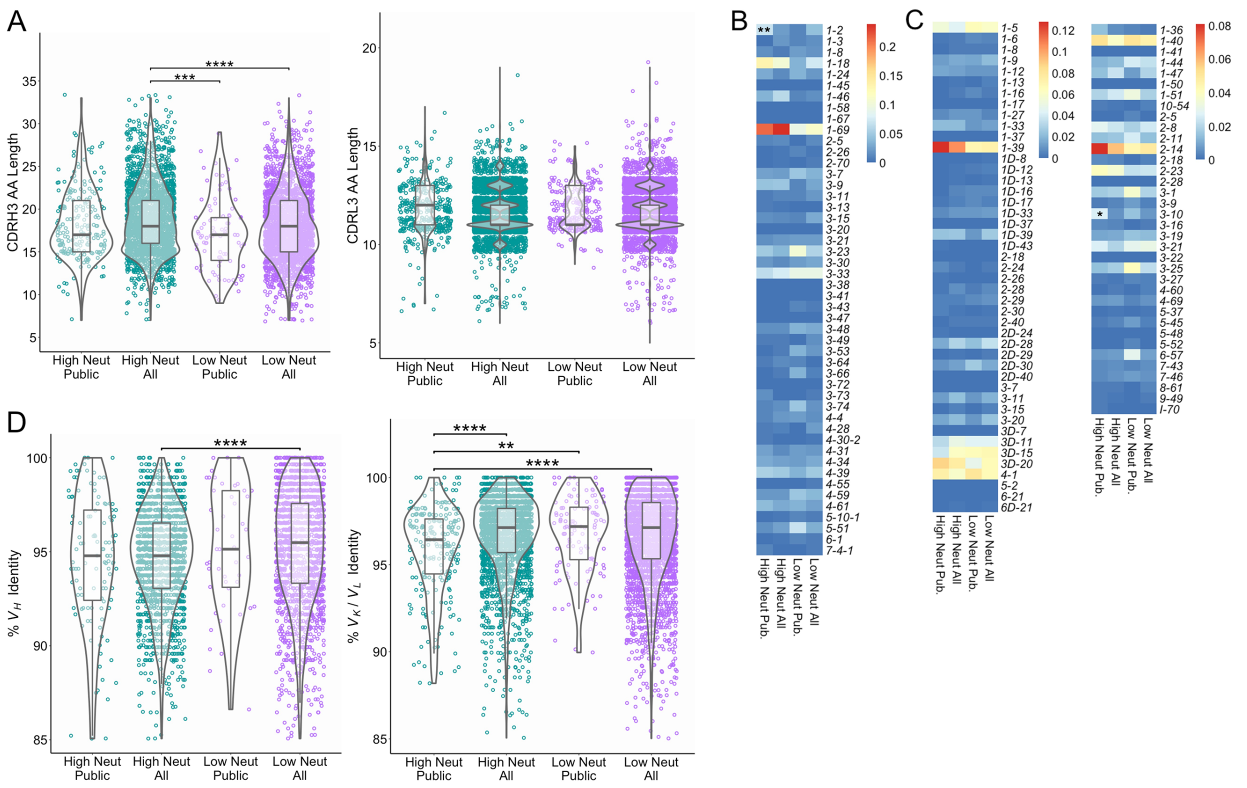

Supplemental Figure S5. BCR characteristics of public clonotypes. (A) CDR3 length of the heavy chain (left panel) and light chain (right panel) is shown for public and non-public B cell clonotypes for high and low neutralization subjects. **(B)** Heatmap showing V_H gene usage for public and non-public B cell clonotypes for high and low neutralization subjects. Statistically significant differences between public and non-public clonotypes for each group (high or low neutralization) are identified with asterisks. **(C)** Heatmap showing V_K (left panel) and V_L (right panel) gene usage for public and non-public B cell clonotypes for high and low neutralization subjects. Statistically significant differences between public and non-public clonotypes for each group (high or low neutralization) are identified with asterisks. **(D)** Somatic hypermutation rates of V_H (left panel) and V_K/V_L (right panel) genes are shown for public and non-public B cell clonotypes for high and low neutralization subjects. Somatic hypermutation is expressed as percent identity to germline V-genes. For **(A)** and **(D)**, violin plots show population distributions. Boxplots indicate the 25th percentile (lower border), 75th percentile (upper border), median (horizontal line), and maximum and minimum values that fall within 1.5x the interquartile range (whiskers). Statistical comparisons for **(A)** and **(D)** were made using the Kruskal-Wallis test followed by the Dunn post-hoc test with the Benjamini-Hochberg correction applied for multiple comparisons. Statistical comparisons for **(B)** and **(C)** were made using Fisher's exact test with the Benjamini-Hochberg correction for multiple comparisons. P_{adj}, adjusted P-value; *, $P < 0.05$; **, $P < 0.01$; *** $P < 0.001$; ****, $P < 0.0001$.
