# Supplementary material for: Convergent antibody responses are associated with broad neutralization of hepatitis C virus
Source: Front Immunol. 2023 Mar 24;14:1135841. doi: 10.3389/fimmu.2023.1135841 (PMC10080129; doi:10.3389/fimmu.2023.1135841)
Supplement: Supplementary file 6 [file Image_6.pdf]

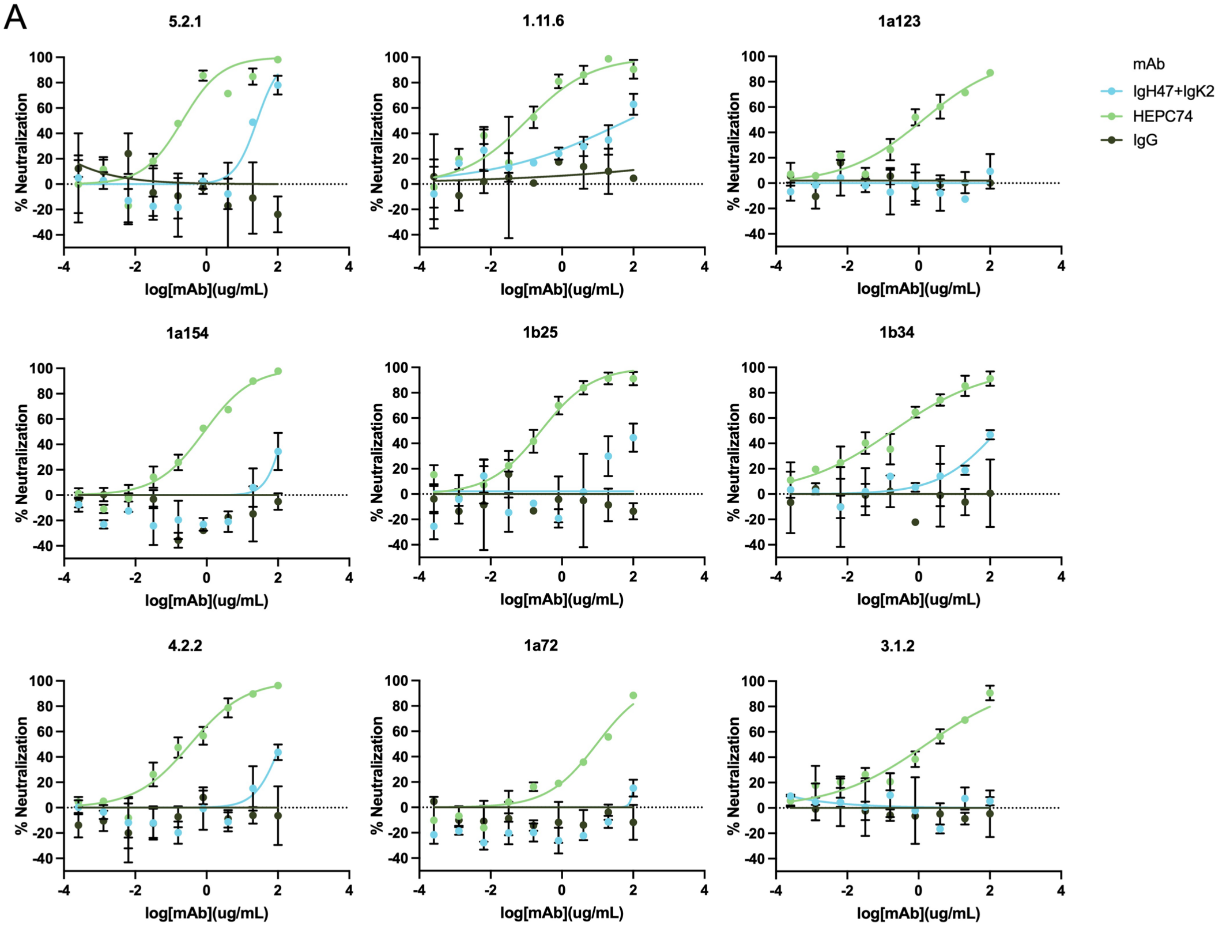

B

HCVpp	IgH57 + IgK2	HEPC74
5.2.1	26.2	0.2
1.11.6	67.0	0.1
1a123	100	1.3
1a154	100	0.8
1b25	100	0.3
1b34	100	0.2
4.2.2	100	0.3
1a72	100	9.5
3.1.2	100	1.6

Supplemental Figure S6. IC_{50} calculation for public clonotype mAb. (A) Percent neutralization of 9 HCVpp by increasing concentrations of IgH47+IgK2. All HCVpp found to have > 20% neutralization at 100 μg/ml (Figure 8) were included. Known bNAb HEPC74 was included as a positive control and non-reactive human IgG was used as a negative control. Values are the average of duplicate wells. **(B)** IC_{50} in μg/mL for each HCVpp tested in (A) calculated from the neutralization curves fit by nonlinear regression (log[inhibitor] vs normalized response, variable slope). mAb-HCVpp tests that did not reach 50% inhibition were assigned an IC_{50} of 100 μg/mL.
