# Supplementary material for: Convergent antibody responses are associated with broad neutralization of hepatitis C virus
Source: Front Immunol. 2023 Mar 24;14:1135841. doi: 10.3389/fimmu.2023.1135841 (PMC10080129; doi:10.3389/fimmu.2023.1135841)
Supplement: Supplementary file 7 [file Table_1.docx]

Supplemental Table 1

| **Subject** | **Sex** | **Age** | **HCV RNA^1^** | **DOI^2^** | **Infection Outcome** | **Neut. Score** | **Neut. Category** |
| --- | --- | --- | --- | --- | --- | --- | --- |
| C48 | M | 27 | 4.9 | 379 | Clearance | 12 | High |
| P53 | M | 30 | 6.5 | 318 | Persistence | 11 | High |
| P54 | M | 28 | 6.5 | 344 | Persistence | 19 | High |
| C117 | F | 31 | 6.3 | 285 | Clearance | 28 | High |
| C172 | F | 21 | 4.4 | 377 | Clearance | 13 | High |
| P49 | M | 30 | 6.5 | 157 | Persistence | 1 | Low |
| P111 | F | 27 | 5.3 | 87 | Persistence | 2 | Low |
| P157 | M | 24 | 5.0 | 376 | Persistence | 2 | Low |
| C178 | F | 25 | 1.4 | 108 | Clearance | 1 | Low |
| C429 | M | 32 | 1.8 | 82 | Clearance | 1 | Low |

1. log_10_ IU/mL

2. Duration of Infection (days)
