# Supplementary material for: Convergent antibody responses are associated with broad neutralization of hepatitis C virus
Source: Front Immunol. 2023 Mar 24;14:1135841. doi: 10.3389/fimmu.2023.1135841 (PMC10080129; doi:10.3389/fimmu.2023.1135841)
Supplement: Supplementary file 8 [file Table_2.docx]

Supplemental Table 2

| **Substitution** | ***IGHV*** | **Prop. Usage** | |
| --- | --- | --- | --- |
|  |  | **High Neut.** | **Low Neut.** |
| **27D** | *1-2* | 0.007 | 0.02 |
|  | *1-3* | 0 | 0.02 |
|  | *1-18* | 0 | 0.07 |
|  | *1-24* | 0.007 | 0.03 |
|  | *1-46* | 0.02 | 0.00 |
|  | *1-69* | 0.56 | 0.20 |
|  | *3-33* | 0 | 0.03 |
|  | *4-4* | 0.007 | 0.07 |
|  | *4-28* | 0.01 | 0.03 |
|  | *4-31* | 0.07 | 0.07 |
|  | *4-39* | 0.21 | 0.20 |
|  | *4-55* | 0 | 0.02 |
|  | *4-59* | 0.04 | 0.13 |
|  | *4-61* | 0.06 | 0.11 |
|  | *5-51* | 0 | 0.02 |
| **29S** | *1-18* | 0.04 | 0 |
|  | *1-46* | 0.04 | 0 |
|  | *1-69* | 0.83 | 0.5 |
|  | *3-7* | 0.04 | 0 |
|  | *4-34* | 0 | 0.25 |
|  | *4-39* | 0.04 | 0.25 |
| **52T** | *1-2* | 0.04 | 0 |
|  | *1-18* | 0.01 | 0.08 |
|  | *1-46* | 0.05 | 0 |
|  | *1-69* | 0.45 | 0.22 |
|  | *3-9* | 0.18 | 0.08 |
|  | *3-11* | 0.01 | 0.03 |
|  | *3-15* | 0 | 0.03 |
|  | *3-20* | 0.01 | 0 |
|  | *3-21* | 0.08 | 0.03 |
|  | *3-23* | 0.04 | 0.17 |
|  | *3-33* | 0.03 | 0.03 |
|  | *3-48* | 0.04 | 0.17 |
|  | *3-53* | 0.01 | 0 |
|  | *3-64* | 0 | 0.03 |
|  | *4-4* | 0.01 | 0 |
|  | *4-34* | 0.01 | 0.03 |
|  | *4-39* | 0.03 | 0.06 |
|  | *4-59* | 0 | 0.06 |
| **52_B_L** | *1-2* | 0.01 | 0.08 |
|  | *1-69* | 0.99 | 0.75 |
|  | *4-39* | 0 | 0.08 |
| **52_B_M** | *1-46* | 0 | 0.08 |
|  | *1-69* | 1 | 0.98 |
|  | *6-1* | 0 | 0.02 |
| **54I** | *1-8* | 0.05 | 0 |
|  | *1-18* | 0.05 | 0.5 |
|  | *1-24* | 0.05 | 0 |
|  | *1-69* | 0.84 | 0.5 |
| **54V** | *1-24* | 0.02 | 0 |
|  | *1-69* | 0.98 | 0.85 |
|  | *3-23* | 0 | 0.08 |
|  | *3-33* | 0 | 0.08 |
| **57T** | *1-69* | 0.92 | 0.63 |
|  | *2-26* | 0 | 0.01 |
|  | *2-5* | 0 | 0.01 |
|  | *3-7* | 0 | 0.09 |
|  | *3-9* | 0.01 | 0.07 |
|  | *3-11* | 0.01 | 0.01 |
|  | *3-21* | 0.01 | 0.01 |
|  | *3-30* | 0 | 0.05 |
|  | *3-33* | 0.02 | 0.05 |
|  | *3-48* | 0.03 | 0.03 |
|  | *6-1* | 0 | 0.03 |
