# Supplementary material for: Convergent antibody responses are associated with broad neutralization of hepatitis C virus
Source: Front Immunol. 2023 Mar 24;14:1135841. doi: 10.3389/fimmu.2023.1135841 (PMC10080129; doi:10.3389/fimmu.2023.1135841)
Supplement: Supplementary file 9 [file Table_3.docx]

Supplemental Table 3

| **Substitution** | ***IGKV/IGLV*** | **Prop. Usage** | |
| --- | --- | --- | --- |
|  |  | **High Neut.** | **Low Neut.** |
| 27D F | *IGKV2-29* | 0 | 0.07 |
|  | *IGKV2-30* | 0.02 | 0 |
|  | *IGKV2D-30* | 0.09 | 0.07 |
|  | *IGKV4-1* | 0.89 | 0.86 |
| 30I | *IGKV1-12* | 0 | 0.03 |
|  | *IGKV1-39* | 0.63 | 0.25 |
|  | *IGKV1-5* | 0.02 | 0.06 |
|  | *IGKV1-9* | 0.02 | 0 |
|  | *IGKV1D-39* | 0.10 | 0.09 |
|  | *IGKV2-24* | 0.02 | 0 |
|  | *IGKV2-40* | 0.02 | 0 |
|  | *IGKV3-15* | 0.02 | 0 |
|  | *IGKV3D-11* | 0 | 0.03 |
|  | *IGKV3D-15* | 0 | 0.03 |
|  | *IGKV3D-20* | 0.03 | 0.09 |
|  | *IGKV4-1* | 0.02 | 0 |
|  | *IGLV1-40* | 0.02 | 0.03 |
|  | *IGLV1-44* | 0.02 | 0.03 |
|  | *IGLV1-47* | 0.02 | 0.09 |
|  | *IGLV1-51* | 0 | 0.06 |
|  | *IGLV3-19* | 0 | 0.06 |
|  | *IGLV3-21* | 0.03 | 0.09 |
|  | *IGLV3-25* | 0.03 | 0.03 |
|  | *IGLV9-49* | 0.02 | 0 |
| 31I | *IGKV1-12* | 0.01 | 0 |
|  | *IGKV1-17* | 0 | 0.02 |
|  | *IGKV1-27* | 0.02 | 0.05 |
|  | *IGKV1-33* | 0.01 | 0.02 |
|  | *IGKV1-39* | 0.47 | 0.12 |
|  | *IGKV1-5* | 0.09 | 0.17 |
|  | *IGKV1-6* | 0 | 0.02 |
|  | *IGKV1-9* | 0.01 | 0.07 |
|  | *IGKV1D-17* | 0 | 0.02 |
|  | *IGKV1D-33* | 0.02 | 0 |
|  | *IGKV1D-39* | 0.03 | 0.02 |
|  | *IGKV2-29* | 0 | 0.02 |
|  | *IGKV2-40* | 0 | 0.02 |
|  | *IGKV2D-28* | 0.03 | 0.02 |
|  | *IGKV2D-30* | 0.02 | 0.02 |
|  | *IGKV3-11* | 0 | 0.02 |
|  | *IGKV3-20* | 0 | 0.02 |
|  | *IGKV3D-11* | 0.02 | 0.02 |
|  | *IGKV3D-15* | 0.08 | 0.10 |
|  | *IGKV3D-20* | 0.05 | 0.10 |
|  | *IGKV4-1* | 0.03 | 0 |
|  | *IGKV6D-21* | 0.01 | 0 |
|  | *IGLV1-44* | 0.01 | 0.02 |
|  | *IGLV2-11* | 0.01 | 0 |
|  | *IGLV2-14* | 0.03 | 0.05 |
|  | *IGLV2-23* | 0 | 0.02 |
|  | *IGLV2-8* | 0.01 | 0 |
