# Supplementary material for: Convergent antibody responses are associated with broad neutralization of hepatitis C virus
Source: Front Immunol. 2023 Mar 24;14:1135841. doi: 10.3389/fimmu.2023.1135841 (PMC10080129; doi:10.3389/fimmu.2023.1135841)
Supplement: Supplementary file 10 [file Table_4.docx]

Supplemental Table 4

| **Sequence ID** | **CDR3 AA Sequence** | **V-gene** | **J-gene** | **% Germline V-gene Identity** | **Nucleotide Sequence** |
| --- | --- | --- | --- | --- | --- |
| IGH-47 | CARAPGITAPDHNWFDPW | *IGHV1-69* | *IGHJ5* | 92.0 | CAGGTGCAGCTGGTGCAGTCTGGGCCTGCGGTGAAGAAGCCTGGGTCCTCGGTGAAGGTATCCTGCCAGGCTTCTGGAGGAACCTTCAGCAGAGATGCTATTAACTGGGTGCGACAGGCCCCTGGACAAGGGCTTGAGTGGATGGGACGGATCATCCCTATTTTTGGCAGACCAAAATACGCACAGAAGTTCCAGGGCAGAGTCACGATTACCGCGGACGAGTCCACGAGCACAGCCTACATGGAGTTGAGCGGCCTGACATCCGAAGACACGGCCGTTTATTATTGTGCGAGAGCCCCGGGTATAACTGCACCTGATCACAATTGGTTCGACCCCTGGGGCCAGGGGACCCTGGTCACCGTCTCCTCAG |
| IGK-2 | CQQSFSAPFTF | *IGKV1-39* | *IGKJ3* | 94.3 | GACATCCAGATGACCCAGTCTCCATCCTCCCTGTCTGCATCTGTAGGAGACAGAGTCACCATCACTTGCCGGGGGAGTCAGACCGTTAGCAGCTATTTAAATTGGTATCAGCAGAAACCAGGGGAAGCCCCTAAACTCCTGATCTATACTGCATCGAGTTTGCAAAGTGGGGTCCCAACAAGGTTCAGTGGCTTTGGATTTGGGACAGATTACACTCTCACCATCACCAATCTGCAACCTGAAGATTTTGCAACTTACTACTGTCAACAGAGTTTCAGTGCCCCCTTCACTTTCGGCCCTGGGACCAAAGTGGATATCGAGC |
